# Tobacco smoke exposure is an independent predictor of vitamin D deficiency in US children

**DOI:** 10.1371/journal.pone.0205342

**Published:** 2018-10-08

**Authors:** Benjamin Udoka Nwosu, Philip Kum-Nji

**Affiliations:** 1 Division of Endocrinology, Department of Pediatrics, University of Massachusetts Medical School, Worcester, Massachusetts, United States of America; 2 Department of General Pediatrics, Children’s Hospital of Richmond, Virginia Commonwealth University School of Medicine, Richmond, Virginia, United States of America; Medical University of Gdańsk, POLAND

## Abstract

**Importance:**

The role of tobacco-smoke exposure on serum vitamin D concentration in US pediatric population is not known. We hypothesized that tobacco smoke exposure would increase the prevalence of vitamin D deficiency in US children.

**Methods:**

Representative national data were accessed from the National Health and Nutrition Examination Survey (NHANES) 2009–2010 databank on 2,263 subjects of ages 3 to 17 years. Subjects were categorized into two groups based on their age: children, if <10 years; and youth if 10 to 17 years. Descriptive and multiple logistic regression analyses were conducted to determine the effect of serum cotinine-verified tobacco smoke exposure on vitamin D status after controlling for key sociodemographic confounders. Vitamin D deficiency was defined as 25(OH)D <20 ng/mL, insufficiency as 25(OH)D of 20–29.9 ng/mL, and sufficiency as 25(OH)D of ≥30 ng/mL. Tobacco smoke exposure status was defined by serum cotinine concentration as follows: unexposed and non-smoking (<0.05 ng/mL) and exposed (passive and active smokers combined) (≥0.05ng/mL). Specifically, passive and active smoking were defined as cotinine of 0.05–10 ng/mL, and ≥10ng/mL respectively.

**Results:**

The prevalence of second-hand smoke exposure was 42.0% (95%CI, 36.7%-47.5%); while the prevalence of active smoking among teenagers was 9.0% (95%CI, 6.2%-12.5%). Vitamin D deficiency occurred at a frequency of 15.1% in children unexposed to tobacco smoke, 20.9% in children exposed to passive tobacco smoke, and 18.0% among actively smoking youth (p<0.001). Tobacco smoke exposure independently predicted vitamin D deficiency after controlling for age, sex, race, BMI, maternal education, and family socio-economic status (OR:1.50; 95%CI, 1.14–1.85, p = 0.002).

**Conclusions:**

This analysis of a nationwide database reports that tobacco smoke exposure is an independent predictor of vitamin D deficiency in US children.

## Introduction

Tobacco smoke exposure in children has been linked to illnesses such as upper and lower respiratory tract infections[1, 2], chronic lung diseases[3, 4], atherosclerosis[5, 6] and sudden infant death syndrome[7], but little is known about the impact of tobacco smoke exposure on vitamin D status in US children and adolescents.

This is important as vitamin D sufficiency is crucial for optimal bone health throughout life[8]. Vitamin D is the principal promoter of bone mineralization, which is the process of depositing calcium and phosphate in osteoid matrix for either bone repair or the formation of new bones[8, 9]. Vitamin D is particularly crucial during the period of growth in children and adolescents for optimal bone mineralization for the attainment of peak bone mass necessary for healthy bones throughout life[10, 11]. Vitamin D sufficiency is also crucial in growing children and adolescents for the extra-skeletal functions of vitamin D such as its improvement of glycemic control through the augmentation of insulin production[12], and the reduction of fasting plasma glucose, hemoglobin A1c, and insulin resistance[13]; improvement in cardiovascular function through the augmentation of myocardial contractility[14]; augmentation of both innate and adaptive immune systems through the enhancement of T_H_2 cell responses by jointly inhibiting T_H_1 cells and stimulating the differentiation of naïve T-cells into T_H_2 cells[15, 16].

However, a recent national report showed that 70% of US children and adolescents have suboptimal vitamin D status[17]. Specifically, 9% had vitamin D deficiency, and 61% had vitamin D insufficiency [17]. This high prevalence of suboptimal vitamin status suggest that a majority of US children and adolescents are at an increased risk for the deleterious effects of vitamin D deficiency or insufficiency which ranges from increased risk for metabolic bone diseases to organ-system dysfunction[18–20].

The risk factors for vitamin D deficiency in US children were reported in a nationwide study in 2009[17], and in another study of inner-city youth in 2012[21], but the impact of tobacco smoke exposure on the vitamin D status of children and adolescents was not addressed in either report and is still not known. Studies in adult subjects have reported that tobacco smoke exposure decreases the serum concentrations of both parathyroid hormone and vitamin D leading to poor absorption of calcium from the gastrointestinal tract and an acceleration of bone loss[22–27]. These findings in adults were however not replicated in a nationwide study of 2515 children and adolescents of 10–18 year old in South Korea which found no relationship between urinary cotinine-verified prevalence of smoking and vitamin D deficiency[28]. The lack of data on tobacco smoke exposure and its impact on vitamin D status in US children means that even when all the known risk factors for vitamin D deficiency are addressed in this population, the unknown risk from tobacco smoke exposure remains. This is rather concerning especially in homes or residential facilities with adult smokers where children are regularly exposed to second hand smoke.

Therefore, we designed this study to assess the relationship between tobacco smoke exposure and vitamin D status in US children and adolescents using a nationally-representative data sample from the National Health and Nutrition Examination Survey (NHANES) 2009–2010 databank. Smoking status was quantified using serum cotinine, the primary proximate metabolite of nicotine, and the gold-standard marker for tobacco smoke exposure[29]. The study’s hypothesis was that tobacco smoke exposure would increase the prevalence of vitamin D deficiency in US children and adolescents. The aim of the study was to determine the relationship between cotinine-verified tobacco smoke exposure and serum 25-hydroxyvitamin D [25(OH)D] concentration in US children and adolescents.

## Subjects and methods

### Ethics statement

The NHANES data collection procedure and protocol were approved by the Centers for Disease Control and Prevention (CDC). All subjects’ records were anonymized and de-identified prior to analysis.

#### Design and study population

This analysis was based on the data from 2009 to 2010 NHANES database[30]. The NHANES is a comprehensive research assessment of health and nutritional status of children and adults in the United States. Data are collected every 2 years through candidate interview, physical examination, and laboratory tests[30, 31].

The NHANES uses stratified cluster complex sampling techniques for its data collection as recommended by the CDC. NHANES protocol oversamples certain population groups in its data collection procedures in order to obtain more accurate and representative information on subgroups that have not been adequately studied in previous examinations. Full details of the complex sampling procedures have been described elsewhere[31].

#### Study variables

During this study, subjects were interviewed at home to obtain detailed socio-demographic information of all household members. Pertinent demographic data include age of subject, sex (male or female), race/ethnicity, height, weight, body mass index (BMI), maternal educational achievement, yearly household family income, and tobacco smoke exposure. In addition, subjects were asked to provide blood samples, in Mobile Examination Centers to determine their serum cotinine and 25(OH)D levels. Serum cotinine levels were measured by an isotope dilution-high performance liquid chromatography[32, 33], while 25(OH)D was measured by ultra-high-performance liquid chromatography-tandem mass spectrometry. Details of the 25(OH)D assay methodology have been described elsewhere[34]. Variables included in the analysis were demographic data, serum cotinine and 25(OH)D concentrations.

#### Definition of terms

Tobacco smoke exposure was quantified based on serum cotinine concentration as follows: cotinine level of <0.05 ng/mL was defined as unexposed or non-smoker; 0.05–10 ng/mL was defined as exposed but not an active smoker (i.e., second-hand smoke or SHS), while >10 ng/mL was defined as an active smoker (AS)[35–37].

Vitamin D deficiency was defined as 25(OH)D of <20ng/mL; vitamin D insufficiency as 25(OH)D of 20–29.9 ng/mL and vitamin D sufficiency as 25(OH)D of ≥30 ng/mL[38]. BMI was calculated by standard method of weight in kg divided by height in meter squared; and was expressed in kg/m^2^ standardized by age and sex. As a standard approach in pediatric studies, calculated BMI values were expressed as percentiles for the assessment of normal-weight-, overweight-, and obesity status as follows: normal-weight (BMI <85^th^ percentile), overweight (BMI >85^th^ but <95^th^ percentile), and obesity (BMI ≥95^th^ percentile).

Only subjects of ages 3–17 years were included in the study. Children of <3 years were excluded because cotinine levels were not available in this age group in the NHANES database, and adolescents of ≥18 years were considered adults. Subjects were categorized into 4 groups: 3 to 5 years, 6 to 9 years, 10 to 14 years, and 15–17 years. Subjects of ages 13 to 17 years were considered to be teenagers. However, for the purposes of simplicity, age was dichotomized into two groups of <10 years (preadolescence or children) and >10 years (adolescence or youth) in the multivariable logistic regression analyses.

#### Statistical analysis

The study’s outcome variable of interest was vitamin D deficiency as indicated by 25(OH)D of <20 ng/dL[38]. The primary independent variable of interest was tobacco smoke exposure as objectively measured by serum cotinine concentration. Other sociodemographic variables that were explored were the age of the respondent, sex, race, maternal education, anthropometric measures (BMI), annual household income, and tobacco smoke exposure.

Descriptive statistics were conducted to determine variables associated with vitamin D deficiency. Chi square test was used to compare the proportions of subjects with 25(OH)D of <20 ng/mL (vitamin D deficiency) by the various demographic variables studied. Student's t-test was used to compare mean serum 25(OH)D concentrations among the categories of selected sociodemographic variables. Multiple logistic regression analysis was further conducted to determine if tobacco smoke exposure was still predictive of vitamin D deficiency after controlling for the other sociodemographic variables. Two categorizations of tobacco smoke exposure were used in separate weighted regression analyses: the first regression analysis was based on the categorization of tobacco smoke exposure into two groups: unexposed (cotinine <0.05ng/mL) and exposed (cotinine level ≥0.05 ng/mL). In a follow-up regression analysis, tobacco smoke exposure was categorized into 3 groups: unexposed, passive smoke exposure (cotinine level 0.05–10 ng/mL), and active smoking (cotinine levels ≥10 ng/mL). In the rest of the multiple regression analyses, the variables were dichotomized as follows: age of child (<10 years vs. ≥10 years); sex (male vs. female); tobacco smoke exposure (exposed vs. not exposed); annual family income (median income of <$55,000 vs. ≥$55,000); maternal education (below college education vs. some college education); race (white vs. non-white); and anthropometrics [normal-weight (BMI <85^th^ percentile vs. overweight/obese (BMI ≥85^th^ percentile)]. We chose a cut-off of 85^th^ percentile to dichotomize the subjects into normal-weight vs. overweight/obese as adiposity is associated with vitamin D deficiency, so the overweight/obese groups could easily be compared to the normal-weight group. Similarly, we grouped the subjects into <10 years or ≥10years as the adolescent years are associated with higher tobacco-smoke exposure given the high-risk behaviors associated with this age group compared to the preadolescent children.

As stated above in the Methods section, some population subgroups were over-sampled for the purposes of maintaining parity in the NHANES database. Therefore, to obtain unbiased national estimates that is representative of the United States population, the present analysis was performed using the complex sample analysis software of the IBM SPSS Statistics for Windows, Version 24.0, Armonk, NY. A p-value of <0.05 was considered statistically significant in all cases.

## Results

### Sociodemographic characteristics and vitamin D deficiency

The subjects consisted of 2,263 children and adolescents of ages 3 to 17 years, with a mean age of 10.2 ± 4.3 years, with 1181 (52%) male subjects. The overall prevalence of suboptimal vitamin D status [25(OH)D of <30 ng/mL] was 64% (95% CI, 58–69). Of this number, 17% (95% CI, 14–22%) had vitamin D deficiency [25(OH)D of <20 ng/mL], and 46% had vitamin D insufficiency [25(OH)D of 20–29.9 ng/mL]. Vitamin D deficiency was more prevalent in the overweight/obese youth (44%) than the normal-weight subjects (15%) (Table 1).

**Table 1:** Prevalence of vitamin D deficiency by sociodemographic characteristics in US children and adolescents.

Parameters	Weighted % of subjects with vitamin D deficiency (95% CI)	p value
All subjects (N = 2263)	17 (13–22)	
Age Group		
<10 (n = 1261)	8 (6–11)	
≥10 (n = 1002)	24 (19–31)	<0.001
Sex		
Male (n = 1181)	14 (10–19)	
Female (n = 1082)	21 (17–26)	<0.001
Race/Ethnicity		
Non-Hispanic White (n = 716)	6 (4–9)	
Mexican American (n = 672)	26 (21–32)	
Other Hispanics (n = 275)	21(13–32)	
African American (n = 444)	46 (35–57)	
Other (n = 156)	28 (15–46)	<0.001
Maternal Education***		
Some college education (n = 1188)	14 (10–18)	
No college education (n = 1013)	22 (17–28)	<0.001
Overweight/Obese (BMI ≥85^th^ percentile) ***		
Yes (n = 871)	29 (22–36)	
No (n = 1369)	15 (11–19)	<0.001
Annual household income ($)***		
≥55,000 (n = 1353)	10 (7–15)	
<55,000 (n = 712)	17 (13–23)	<0.001
Tobacco smoke exposure*		
No exposure (n = 1291)	15 (11–20)	
Exposure (SHS and AS) (n = 1002)	21 (16–26)	0.003
Tobacco smoke exposure**		
No exposure (n = 1261)	15 (11–20)	
Exposed only (SHS) (n = 929)	21 (16–27)	
Actively smoking (AS) (n = 73)	18 (11–29)	0.02

SHS second hand smoke; AS actively smoking.

* composite comparison.

** individual comparison.

***some missing information in this category.

CI = confidence interval.

When compared to patients with 25(OH)D of >20 ng/mL, those with vitamin D deficiency, i.e., 25(OH)D of <20 ng/mL had higher values for weight z score: 0.63 ± 0.04 vs. -0.06 ± 0.02, p <0.001; height z score, 0.56 ± 0.04 vs. -0.02 ± 0.02, p <0.001; and BMI z score 0.58 ± 1.2 vs. 0.08 ±0.9, p < 0.001. Table 1 further shows that female subjects were more likely to be vitamin D deficient than male subjects (p<0.001); and subjects from ethnic minorities were more likely to be vitamin D deficient compared to whites (p<0.001); while subjects from lower income groups were more likely to be vitamin D deficient than their more affluent peers (p<0.001); and finally, offspring of mothers with less education were more likely to be vitamin D deficient compared to offspring of more educated mothers (p<0.001).

### Vitamin deficiency in relation to tobacco smoke exposure

**Fig 1:**
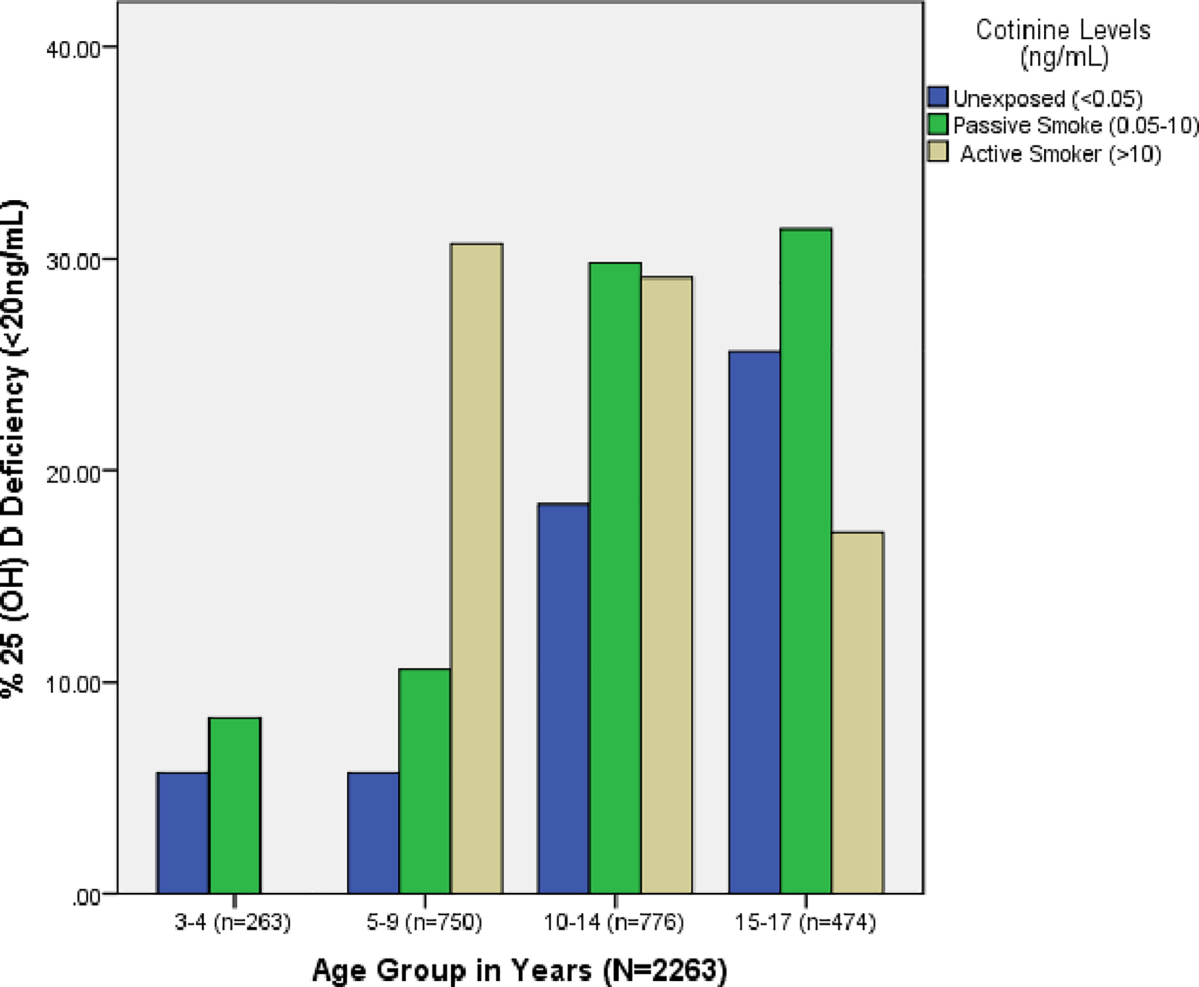
Percentage of US children and adolescents of 3–17 years with vitamin D deficiency stratified by age as well as tobacco-smoke exposure status based on serum cotinine concentration. Subjects with cotinine level of <0.05 ng/mL were characterized as unexposed or non-smokers; those with levels of 0.05–10 ng/mL were characterized as exposed but not active smokers (i.e., second-hand smoke or SHS), while those with levels >10 ng/mL were characterized as active smokers (AS)[35–37]. Passive smoke exposure increased the prevalence of vitamin D deficiency across all age groups, whereas active smoke exposure impacted younger subjects (<15 years) more than their older peers (15-17years).

In US children and adolescents, the prevalence of second hand smoke exposure was 42% (95% CI, 37% - 48%); while the prevalence of serum cotinine concentration in the active smoking range of ≥10 ng/dL was 9% (95% CI, 6–13%) among US teenagers of 13–17 years old. Based on cotinine-verified tobacco-smoke exposure, vitamin D deficiency occurred at a frequency of 15% in unexposed children, 21% in exposed children, and 18% among actively smoking youth (p<0.001) (Table 1) (Fig 1).

Table 2 shows the mean 25(OH)D concentration with the standard error of the mean (SEM) stratified by sociodemographic variables. There was a statistically significant decrease in serum 25(OH)D concentration with increasing tobacco smoke exposure status. Subjects with serum cotinine concentration in the active smoking range had the lowest 25(OH)D concentration compared to the unexposed subjects or passive smokers (p<0.001). All the other selected variables were equally predictive of vitamin concentration.

**Table 2:** Weighted means of serum 25-hydroxyvitamin D concentration in US children and adolescents stratified by confounding variables.

Variable	Mean 25(OH)D (ng/mL) ± SEM	p value
All (N = 2263)	27.8 (0.61)	
Age group (years)		
<10 (n = 1013)	30.2 (0.65)	
≥10 (n = 1250)	26.2 (0.65)	<0.001
Sex		
Male (n = 1012)	28.3 (0.60)	
Female (n = 1250)	27.3 (0.69)	<0.001
Race/Ethnicity		
Non-Hispanic white (n = 715)	31.6 ((1.01)	
Non-white (n = 1547)	23.2 (0.51)	<0.001
Body mass index (kg/m^2^)		
Normal-weight (n = 1368)	28.9 (0.70)	
Overweight/obese (n = 871)	26.0 (0.61)	<0.001
Maternal education		
No college education (n = 1188)	26.0 (0.59)	
Some college education (n = 1003)	29.2 (0.78)	<0.001
Serum cotinine concentration (ng/mL)		
<0.05 (unexposed, n = 1261)	28.1 (0.77)	
0.05–10.0 (passive smoker exposure, n = 928)	27.6 (0.82)	
>10.0 (active smoker, n = 73)	26.7 (1.45)	<0.001

25(OH)D = 25hydroxyitamin D; SEM standard error of the mean

### Multivariate regression analysis of factors associated with vitamin D deficiency

Multiple logistic regression analysis demonstrated that tobacco smoke exposure was predictive of vitamin D deficiency after controlling for anthropometric and socio-demographic confounders such as age, race, BMI, maternal education, and family socio-economic status (OR = 1.5; 95%CI, 1.14–1.85) (p = 0.002) (Table 3). Other independent predictors of vitamin D deficiency, 25(OH)D of <20 ng/mL, in this sample included race, age, sex, and BMI (Table 3). For example, non-white subjects were >8 times more likely to be vitamin D deficient than white subjects, OR = 8.3, (95% CI, 5.69–12.09), while children of >10yr were 5 times more likely to be vitamin D deficient than their younger counterparts of 3–9 years, OR = 4.5 (95% CI, 3.55–6.04). Interestingly, the prevalence of tobacco smoke exposure increased with the age of the subjects, as indicated by an interaction effect between tobacco smoke exposure and the age of the child (p = 0.02), suggesting that tobacco smoke exposure could partly explain the lower serum 25(OH)D in the older subjects. However, in a separate regression analysis (Table not shown), when tobacco smoke exposure was categorized into the 3 groups of no exposure, second hand smoker, and active smoker, tobacco smoke exposure was only predictive of vitamin D deficiency when passive smokers (cotinine level of 0.05–10 ng/mL) were compared to their unexposed counterparts (cotinine levels <0.05 ng/mL); (OR = 1.5, 95% CI 1.18–1.89). In contrast, there was no significant difference between the active smoking group (cotinine levels >10 ng/mL) as compared to those unexposed (OR = 1.14; 95% CI = 0.53–2.48), and the age* cotinine interaction was also non-significant.

**Table 3:** Multiple logistic regression of factors predictive of vitamin D deficiency among children and adolescents of 3–17 years in the United States.

Parameters	Adjusted OR (95% CI)	p value
Race: (non-white vs. white)	8.3 (5.7–12.1)	**<0.001**
Age (years): (≥10 vs. <10)	4.6 (3.6–6.0)	**<0.001**
Sex (female vs. male)	1.9 (1.5–2.4)	**<0.001**
BMI: (overweight/obese vs. normal-weight)	1.7 (1.3–2.2)	**<0.001**
Tobacco smoke exposure vs. non-exposure	1.5 (1.1–1.9)	**0.002**
Annual family income: ($) <55,000 vs >55,000	1.2 (0.9–1.7)	0.14
Maternal education: (no college vs. college education)	1.2 (0.9–1.5)	0.23
Age*Cotinine	1.9 (1.1–3.2)	**0.02**

BMI = body mass index; OR = odds ratio; CI = confidence interval; significant p values are bolded

## Discussion

This is the first nationwide study to characterize the impact of tobacco smoke exposure on the vitamin D status of US children and adolescents. This study’s central finding is that tobacco smoke exposure is associated with an increased risk for vitamin D deficiency in US children and adolescents. This finding adds to the growing list of negative health effects of tobacco smoke exposure in children and adolescents such as upper and lower respiratory tract infections[1, 2], chronic lung diseases[3, 4], atherosclerosis[5, 6] and sudden infant death syndrome[7].

This study reports significant differences in the prevalence of vitamin D deficiency between the groups, with significantly higher prevalence of vitamin D deficiency occurring in female subjects, older youth, overweight/obese subjects, individuals from families of lower socioeconomic status, as well as children and adolescents from ethnic minority groups. These findings are in concert with previous reports [17, 28, 39]. There are several reasons for these findings: (a) the higher prevalence of vitamin D deficiency in female subjects and the overweight/obese subjects has been reported to result from either volumetric dilution, or the sequestration of vitamin D in fat depots in these subjects[40], (b) parents of children and adolescents from families of lower socioeconomic status may not have the financial resources for an optimal vitamin D supplementation regimen for their children; and (c) the darker skin pigmentation in children and adolescents from ethnic minority groups limits the penetration of ultra-violet radiation into the skin for an optimal endogenous vitamin D synthesis.

The prevalence of vitamin D deficiency was also influenced by the overall smoke exposure patterns and the age range of the subjects. Children and adolescents affected by second hand smoke had higher prevalence of vitamin D deficiency compared to active smokers (Fig 1). This finding is similar to a previous report from Korea [28], and was explained by Byun et al[28] as resulting from the association of active smoking with increased exposure to sunlight as it occurs outdoors, while passive smoking occurs mostly indoors with limited exposure to sunlight. Fig 1 further shows that while passive smoke exposure increased the prevalence of vitamin D deficiency across all age groups, active smoke exposure had a greater negative impact on the vitamin D status of younger subjects of <15 years compared to the vitamin D status of their older peers of >15-17years. This stronger effect of passive smoking (which occurs indoors) on the prevalence of vitamin D deficiency over active smoking (which occurs outdoors) was also shown by the attenuating effect of increasing age of subjects on the predictive model of vitamin D deficiency by tobacco smoke exposure in older, actively smoking youth who are mostly outdoors. In summary, the synergistic impact of both passive and active tobacco smoke exposure on the prevalence of vitamin D deficiency is strongest in younger children and adolescents of <15 years.

The high prevalence of suboptimal vitamin D of 64% reported in this study is similar to the 70% reported by Kumar et al[17] in US children and adolescents in 2009, but is lower than the 98% prevalence reported in Korean children and adolescents[28]. The higher prevalence of vitamin D deficiency in Korean children and adolescents compared to their peers in the US may not be due to differences in the magnitude of solar radiation as both countries are located close to latitude 38ºN, but may be due to the comparatively darker skin pigmentation of the majority of Korean youth compared to the lighter skin pigmentation of the majority of US youth who are non-Hispanic white.

Prolonged periods of tobacco smoke exposure in children and adolescents and the attendant high prevalence of suboptimal vitamin D concentrations have health implications as vitamin D has important roles for both skeletal[10, 11] and extra-skeletal health[12–16]. For instance, vitamin D deficiency induces secondary hyperparathyroidism, which in turn increases the activity of osteoclasts compared to osteoblasts resulting in a state of high bone turnover and bone loss[8, 41]. Longstanding periods of vitamin D deficiency leads to poor mineralization of osteoid matrix and consequent development of rickets in children with open epiphyses, or osteomalacia in older youth with closed epiphyses[8]. This physiological derangement resulting from vitamin D deficiency could be exacerbated in individuals exposed to tobacco smoke, as shown in this study, through the process of nicotine induction of hypoparathyroidism[24, 42]. Nicotine activates nicotine receptors in the parathyroid glands resulting in the downregulation of the activities of the glands and consequent hypoparathyroidism[24, 42]. This nicotine-induced hypoparathyroidism is supported by studies reporting reduced serum 1,25-dihydroxyvitamin D concentration, along with subnormal parathyroid hormone concentration, and elevated serum phosphorus in smokers[24, 43, 44]. This downregulation of the parathyroid gland function could explain the reported deleterious effect of tobacco smoke exposure on bone in animals [45, 46] and humans[23, 47], as parathyroid hormone is the primary factor that activates the enzyme, 1α-hydroxylase, which converts 25(OH)D to the biologically active form, 1,25-dihydroxyvitamin D. This biologically active form of vitamin D, in turn, increases the absorption and reabsorption of both calcium and phosphorus from the intestine and kidney respectively[8]. This study suggests that these deleterious effects of tobacco smoke exposure on vitamin D concentration are more pronounced in female subjects, older youth, overweight/obese subjects, individuals from families of lower socioeconomic status, as well as children and adolescents from ethnic minority groups.

Taken together, tobacco smoke exposure may adversely affect mineral metabolism by downregulating parathyroid gland activity and impairing the 1-α-hydroxylation of 25(OH)D to form 1,25-dihydroxyvitamin D.

This study has several limitations which should be taken into consideration in the interpretation of the results. The cross-sectional design of the study precludes causality. We did not have data on subjects’ biochemical parameters such as parathyroid hormone, calcium, phosphorus, 1,25-dihydroxyvitamin D, as well as non-biochemical determinants of vitamin D status such as seasons, dietary and supplemental vitamin D intake. The availability of these biochemical parameters could have allowed us to demonstrate evidence for vitamin D deficiency-related hyperparathyroidism, as well as related changes in calcium, phosphorus, and the active form of vitamin D, 1,25-dihydroxyvitamin D. The availability of data on season of vitamin D collection and dietary supplement history would have enabled us to further adjust our results for these variables, and to determine if there were differences in vitamin D supplementation between the higher and lower socioeconomic groups. The strengths of this study include the representative sample of US children and adolescents across a broad age range; large sample size with rigorous data collection protocol; the use of an objective marker, serum cotinine, to quantify tobacco smoke exposure; and the measurement of serum vitamin D with a state-of-the-art technique.

## Conclusion

This analysis of a nationwide database reports that tobacco smoke exposure is an independent predictor of vitamin D deficiency in US children. This finding is important for public health policies directed at improving the vitamin D status of children and adolescents in the US.
